# A Terrain-Constrained TIN Approach for High-Precision DEM Reconstruction Using UAV Point Clouds

**DOI:** 10.3390/jimaging12010008

**Published:** 2025-12-25

**Authors:** Ziye He, Shu Gan, Xiping Yuan

**Affiliations:** 1 Faculty of Land Resource Engineering, Kunming University of Science and Technology, Kunming 650093, China; hzy@stu.kust.edu.cn; 2 Application Engineering Research Center of Spatial Information Surveying and Mapping Technology in Plateau and Mountainous Areas Set by Universities in Yunnan Province, Kunming 650093, China

**Keywords:** UAV point cloud, digital elevation model, triangulated irregular network interpolation, surface roughness, complex terrain

## Abstract

To address the decline in self-consistency and limited spatial adaptability of traditional interpolation methods in complex terrain, this study proposes a terrain-constrained Triangulated Irregular Network (TIN) interpolation method based on UAV point clouds. The method was tested in the southern margin of the Lufeng Dinosaur National Geopark, Yunnan Province, using ground points at different sampling densities (90%, 70%, 50%, 30%, and 10%), and compared with Spline, Kriging, ANUDEM, and IDW methods. Results show that the proposed method maintains the lowest RMSE and MAE across all densities, demonstrating higher stability and self-consistency and better preserving terrain undulations. This provides technical support for high-precision DEM reconstruction from UAV point clouds in complex terrain.

## 1. Introduction

Digital Elevation Models (DEMs) serve as fundamental data for terrain analysis [1], hydrological modeling [2], and ecological environment assessment [3], and their accuracy directly affects the effectiveness of these applications. To generate high-precision DEMs, raw point cloud data typically undergo filtering [4], ground point classification [5], and spatial interpolation [6]. Conventional interpolation methods, such as inverse distance weighting (IDW) [7], ordinary Kriging [8], and spline functions [9], usually apply a uniform interpolation model and parameters across the entire study area. This “one-size-fits-all” approach has inherent limitations because it overlooks the spatial non-stationarity of surface processes, meaning that terrain morphology and its statistical properties vary spatially.

Adaptive interpolation refers to a class of algorithms that dynamically adjust their interpolation strategies, model parameters, or kernel functions according to local terrain features or data distribution characteristics. The core idea is to recognize and quantify terrain heterogeneity, thereby matching the interpolation process to the complexity of local surface processes. From a geostatistical perspective, adaptive interpolation can be regarded as a response to spatial non-stationarity.

For example, Li et al. [10] proposed a method that employs three hierarchical provisional digital elevation model (DEM) raster surfaces combined with thin plate spline (TPS) interpolation to separate ground points from unclassified points using adaptive residual thresholds. At the same time, a cloth simulation algorithm is applied to generate sufficient effective initial ground seeds, enabling the construction of high-quality topographic surfaces. Wang et al. [11] proposed a DEM construction strategy combining high-accuracy surface modeling (HASM) with classical interpolation. TINs are built from original terrain data, and supplementary points (SA-Points) are added based on triangle areas. Elevations for SA-Points are interpolated using IDW, Kriging, or spline methods, and then merged with original data to generate the DEM. Arnone [12] employed the distributed eco-hydrological-landslide model tRIBS-VEGGIE-Landslide to assess the impact of terrain resolution on hydro-geomorphological processes in slope stability analysis. The model describes the topography using a TIN derived from a grid-DEM. The study compared the performance of deterministic interpolation methods IDW, Natural Neighbor (NN) and the geostatistical method (OK) in generating DEMs and quantifying terrain roughness. The results indicate that DEM quality is closely related to roughness values, with the NN method showing the best performance in terms of accuracy and standard deviation [13].

Terrain surface roughness (TSR) is an important topographic parameter in geosciences, and its accuracy is influenced by the DEM generation method and the density of LiDAR data [14,15]. Bui et al. [16] proposed an end-to-end workflow to quantify the uncertainty of LiDAR-derived DEMs without requiring independent validation data. The method uses per-point observation uncertainties as input, computes interpolation-induced propagated uncertainties via GLOPOV, and scales them according to point density and terrain roughness. Fan et al. [17] compared five commonly used roughness descriptors, analyzing their correlations in quantifying terrain surface roughness maps across three types of terrain with different spatial variations, and investigated the effects of spatial scale and interpolation methods. The results show that while the roughness maps exhibit similar global patterns, local patterns differ, highlighting the importance of using multiple descriptors in local roughness analyses.

Recent advances in adaptive terrain modeling have emphasized locally varying interpolation parameters [18], feature-preserving TIN refinement, and multi-scale representation of topographic heterogeneity. Adaptive TIN techniques [19], breakline-enhanced triangulation, and spatially varying kernel methods have been used to improve DEM generation in heterogeneous landscapes. Studies have also explored local surface fitting [20], and hybrid TIN–grid [21] approaches to better handle abrupt elevation changes and non-stationary terrain processes. Situating the present work within this context, our study contributes a comparative evaluation of a TIN-based refinement strategy under varying UAV point cloud densities, focusing on its behavior relative to widely used deterministic and geostatistical methods.

In this paper, the term DEM is used in its broader sense, encompassing both digital surface models (DSMs) and bare-earth digital terrain models (DTMs). Here we specifically refer to the bare-earth DTM generated after ground point classification and removal of vegetation and man-made objects, which is the standard product required for geomorphological and hydrological analyses.

To address the issues outlined above, this study focuses on three typical mountainous terrain areas as research subjects. Using original point cloud data, sampling is conducted at ratios of 90%, 70%, 50%, 30%, and 10%. An improved TIN interpolation method is proposed and compared with four other commonly used interpolation algorithms to construct DEM data. The objective is to provide theoretical basis and methodological support for the construction and application of high-precision DEMs and their derived topographic parameters. A comprehensive evaluation of the generated DEM products is performed from two dimensions: self-consistency assessment and surface roughness-derived analysis. The main contributions of this study are as follows: We propose a Terrain-Constrained TIN (TC-TIN) interpolation method, integrating adaptive seed-point screening and boundary auxiliary point insertion to improve DEM reconstruction in complex mountainous terrain. We establish a density-degradation evaluation framework using UAV point clouds at five sampling densities (90%, 70%, 50%, 30%, 10%). We conduct a comprehensive comparison between TC-TIN and commonly used interpolation methods including Kriging, IDW, Spline, ANUDEM. We analyze the sensitivity of TC-TIN to key parameters, such as seed neighborhood radius and maximum edge length, and discuss their selection principles.

The remainder of this manuscript is organized as follows. Section 2 describes the study area, data preprocessing, and the proposed TC-TIN interpolation method. Section 3 presents the experimental results, including DEM self-consistency analysis, surface roughness evaluation, and statistical tests. Section 4 discusses the implications, limitations, and comparisons with existing studies. Section 5 concludes the paper and outlines possible directions for future work.

## 2. Materials and Methods

### 2.1. Study Area

The study area is located in the southern mountainous region of the Lufeng Dinosaur National Geopark, Chuxiong Yi Autonomous Prefecture, Yunnan Province, China. It lies within a subtropical low-latitude plateau monsoon climate zone, characterized by distinct wet and dry seasons. The geomorphology is highly diverse and complex, dominated by erosional and mesa landforms. The terrain gradually descends from northeast to southwest, with elevations ranging from 2200 m at the highest point to 1302 m at the lowest, resulting in a pronounced topographic relief.

**Figure 1:**
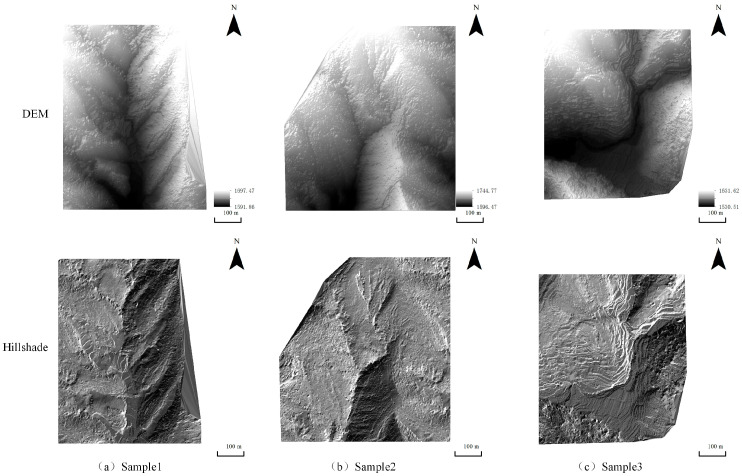
DEM and Hillshade of the Study Area: (**a**) Sample 1; (**b**) Sample 2; (**c**) Sample 3.

The stratification types are also diverse, including horizontal, cross-bedded, graded, wavy, and trough bedding structures. As shown in Figure 1, three representative mountainous geomorphological areas along the southern margin of the Lufeng Dinosaur Valley were selected for this study. These areas encompass erosional hills, fault valleys, and mesa terraces, covering the main morphological features of typical mid-mountain topography in Yunnan Province. Such diversity provides a representative testbed for evaluating the adaptability and limitations of interpolation algorithms under complex terrain conditions. Relevant information on the experimental data is presented in Table 1.

### 2.2. Data Source and Preprocessing

The raw imagery was acquired on 20 December 2024 using a DJI Mavic 3E drone equipped with PPK/RTK positioning. Dense point clouds were generated via Structure-from-Motion (SfM) photogrammetry in Agisoft Metashape Professional (v2.1.1) using high-quality settings and aggressive depth filtering, yielding an average ground point density of approximately 1.5 pts/m^2^ after ground classification and filtering.

The raw point cloud data were denoised and filtered using Terra Solid (version 2022.1) software to extract ground points of the study area. Subsequently, 10% of the ground points were randomly selected as a validation dataset to evaluate the interpolation self-consistency of the generated DEM. To investigate the effect of sampling density, the voxel grid downsampling method was applied to the point cloud data at sampling rates of 10%, 30%, 50%, 70%, and 90%. Regional clipping was then performed to obtain corresponding point cloud subsets for DEM construction. The 10% sampling density was specifically selected for detailed visual and quantitative comparisons because it represents an extreme yet realistic data-sparsity scenario commonly encountered in practical applications (e.g., lower-cost drones, higher flight altitudes, or the need for aggressive down-sampling to reduce computational burden). The point cloud densities under different sampling ratios are presented in Table 2.

### 2.3. Triangulated Irregular Network

TIN models the terrain surface by constructing a mesh of irregular triangles from control points. It fits the ground surface by triangulating discrete point clouds to form a TIN, then assigning elevation values to the triangle vertices to generate a continuous surface. This method accurately adapts to elevation variations in complex terrain, effectively representing the topographic surface in the form of an irregular triangular mesh [22]. The process of generating a DEM using TIN can be divided into three steps.

#### 2.3.1. Discrete Points

Prior to TIN construction, foundational elevation data must be prepared, typically acquired through surveying or remote sensing. During the data preprocessing phase, raw data should undergo systematic cleaning, denoising, and missing value imputation to eliminate outliers, enhance data completeness, and ensure the self-consistency of elevation information. High-quality preprocessing is a critical prerequisite for the self-consistency of subsequent triangulation and interpolation.

#### 2.3.2. Triangulation

Triangulation is the core process of converting discrete elevation points into a continuous terrain surface. The objective is to connect elevation points to form a set of non-overlapping triangles, thereby constructing a complete TIN structure. Common algorithms include Delaunay triangulation, insertion algorithms, and incremental algorithms. Among these, Delaunay triangulation is widely used in high-precision terrain modeling because it ensures that no other vertices lie within the circumcircle of any triangle, thereby maximizing the minimum angle and reducing the occurrence of elongated triangles. This method effectively improves the geometric uniformity and structural stability of the TIN.

#### 2.3.3. Elevation Assignment

After generating the triangular mesh, elevations for locations within the triangles must be calculated to form a continuous DEM surface. The interpolation process estimates the elevation at any location based on the known elevation values at the triangle vertices. Common methods include linear interpolation and polynomial interpolation. Linear interpolation is widely adopted due to its computational simplicity and suitability for regular terrain, while polynomial interpolation can provide higher fitting self-consistency in complex or highly undulating terrain. Appropriate selection of the interpolation method can significantly enhance the spatial continuity and terrain representation capability of the DEM.

The interpolation formula for the TIN is as follows:

(1) $$\begin{array}{c} \hat{Z}\left(x_{0},y_{0}\right)=\alpha Z_{1}+\beta Z_{2}+\gamma Z_{3} \end{array}$$

In this formulation: $\hat{Z}\left(x_{0},y_{0}\right)$ represents the elevation or value of the point to be estimated, $Z_{1}$, $Z_{2}$, and $Z_{3}$ represent the elevations of the vertices of the triangle containing the point, and α, β, and γ represent the barycentric coordinates of the point within the triangle, which are used to linearly combine the vertex elevations to ensure that the interpolated value lies on the triangle plane.

### 2.4. TC-TIN Interpolation Algorithm

Significant differences exist in characteristic metrics such as point cloud density and elevation standard deviation between densely matched point clouds and 3D laser scanning point clouds. If traditional TIN densification algorithms are applied directly for DEM generation, issues often arise, including insufficient self-consistency and registration deviations. These issues subsequently compromise the analysis and application effectiveness of the point cloud data. Furthermore, it is crucial to prevent non-ground points, such as those from buildings and vegetation, from being misclassified as ground points, as this would adversely affect DEM self-consistency.

To address these challenges, this study introduces optimizations and improvements upon the traditional triangulation algorithm. By implementing candidate seed point screening and the introduction of boundary auxiliary points, the rationality of TIN construction and the self-consistency of the generated DEM are enhanced.

#### 2.4.1. Seed Point Screening

In the traditional TIN construction process, some seed points may lie on non-ground surfaces of objects. If these points directly participate in triangulation, they introduce elevation errors into the DEM. To ensure that points involved in the network construction are genuine ground points, this study proposes a seed point screening mechanism based on local elevation comparison. For each candidate point, the algorithm searches its surrounding points within a defined neighborhood. If points with lower elevations are found, the candidate point is identified as a potential non-ground point and is subsequently eliminated. If no lower points exist, the candidate point is retained as a valid ground seed point. This step significantly reduces the impact of misclassified object points and improves the topographic representativeness of the TIN nodes.

In this study, a fixed search radius of 3 m and a minimum-neighbor count of k = 8 were adopted. A candidate point was removed if any neighbor had an elevation at least 0.15 m lower than the candidate. This conservative “single-lower-point rule” effectively filters out points located above the ground surface and improves the reliability of TIN node selection in complex terrain.

#### 2.4.2. Boundary Auxiliary Point Addition

**Figure 2:**
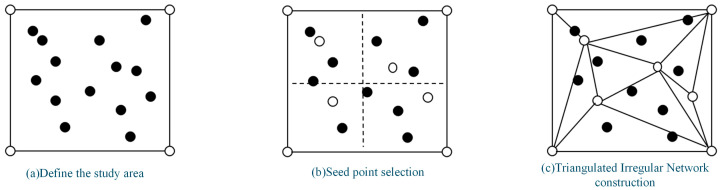
Workflow of Irregular Triangulated Network (TIN) Construction: (**a**) Define the study area; (**b**) Seed point selection; (**c**) Triangulated Irregular Network construction.

To prevent the formation of overly narrow or discontinuous triangles in boundary regions of the TIN, this study introduces an auxiliary point mechanism governed by a maximum edge length constraint (Figure 2). This boundary value serves as an upper limit parameter to control the distribution of triangle edge lengths and the overall mesh morphology. An excessively small boundary value may lead to jagged, discontinuous edges in the TIN, resulting in poor DEM continuity. Conversely, an excessively large value may cause redundant triangulation in boundary areas. Therefore, considering the terrain characteristics and boundary variation patterns, feature-based auxiliary points are judiciously added. This optimizes the triangular mesh structure, ensuring the generated DEM exhibits superior smoothness along its boundaries.

In this study, the maximum edge length was set to 10 m, determined as approximately 8 times the mean point spacing of the study area. When a Delaunay triangle edge exceeded this limit, a boundary auxiliary point was inserted at the midpoint of the edge, with its elevation computed by linear interpolation of the two endpoint elevations. This strategy enhances the stability and continuity of the DEM near boundary regions.

The experimental design of this study is divided into two phases. The first phase focuses on analyzing the impact of different spatial interpolation methods and sampling densities on DEM self-consistency.

First, point cloud data obtained by UAV is used as the original material. After point cloud filtering, ground points are extracted for DEM construction. All ground points are randomly split into modeling dataset and validation dataset at a specified ratio, with 90% of the ground points used as modeling dataset for interpolation modeling, and the remaining 10% reserved as independent validation dataset for subsequent self-consistency assessment.

To investigate the influence of data density on interpolation results, each subset of data is used to generate DEMs by applying five commonly used spatial interpolation methods. These include: the ANUDEM method (Australian National University Digital Elevation Model) [23,24], Inverse Distance Weighting (IDW), Kriging, and Spline interpolation. DEMs at different densities are constructed using the above methods. Subsequently, the reserved 10% of verification points are used to quantitatively assess the accuracy of each DEM. Evaluation metrics include Root Mean Square Error (RMSE) and Mean Absolute Error (MAE) [25], which are employed to measure the elevation fitting accuracy of the different methods.

Ground point filtering and classification were performed using TerraScan in TerraSolid (v21.014). The default ground extraction workflow was used, with minor manual adjustments to remove residual vegetation points. The ANUDEM interpolation was conducted using ANUDEM version 5.3, with drainage enforcement enabled and other parameters set to their recommended defaults.

To further examine the ability of different interpolation methods to recover terrain details, the study also conducts surface roughness analysis. This includes the generation of roughness maps and the calculation of roughness RMSE, among other indicators, to comprehensively evaluate the performance of each interpolation method under complex terrain conditions.

Surface roughness refers to the degree of variation in surface elevation within a certain spatial range, typically quantified using parameters such as the standard deviation of elevation, slope variation, or topographic relief. This paper employs the Root Mean Square Height (RMSH) [26] method to calculate surface roughness. This parameter reflects the degree of elevation fluctuation of the terrain surface.

(2) $$\begin{array}{c} \mathrm{RMSH}=\sqrt{\frac{1}{n-1}\sum_{i=1}^{n}{\left(H_{i}-\bar{H}\right)}^{2}} \end{array}$$

In this formulation, n is the number of pixels within the analysis window. A fixed window size of 3 × 3 pixels (*n* = 9) was adopted throughout this study. $H_{i}$ is the elevation of the i-th pixel in the window, and $\bar{H}$ denotes the mean elevation of all pixels in that window. This metric reflects local topographic variability, with larger RMSH values indicating rougher terrain.

### 2.5. Self-Consistency Assessment

Due to the lack of independent ground truth (e.g., TLS or RTK survey), the validation in this study evaluates interpolation self-consistency rather than absolute accuracy. Owing to the complex topography of the study area and the absence of ground control points, it was infeasible to obtain high-precision elevation data through field surveys, which limited the direct self-consistency evaluation of the DEMs generated from the UAV point cloud. To address this, this study employed a cross-validation approach. Specifically, 10% of the ground point cloud was extracted to serve as a validation sample set for assessing the self-consistency of DEMs produced under different point cloud densities and interpolation methods.

Two widely used quantitative accuracy metrics—Root Mean Square Error (RMSE) and Mean Absolute Error (MAE)—were adopted for the evaluation. Their calculation formulas are given as follows:

(3) $$\begin{array}{c} RMSE=\sqrt{\frac{\sum_{i=1}^{n}{\left(z_{i}-z_{i}^{*}\right)}^{2}}{n}} \end{array}$$

(4) $$\begin{array}{c} MAE=\frac{1}{n}\sum_{i=1}^{n}\left|z_{i}-z_{i}^{*}\right| \end{array}$$

In this formulation, $z_{i}$ represents the actual elevation value of the *i*-th sample point; and $z_{i}^{*}$ represents the predicted elevation value at the *i*-th sample point after interpolation; n represents the total number of sample points.

This method is used to analyze the self-consistency of the interpolated DEM. Higher values of RMSE and MAE indicate greater deviation between the DEM and the actual terrain, corresponding to lower self-consistency.

### 2.6. Statistical Significance Test

To further evaluate whether the differences in RMSE and MAE among the interpolation methods are statistically meaningful, a paired *t*-test was performed. For each study area and sampling density, the RMSE and MAE values generated by the proposed method were paired with those from the Spline and Kriging methods, which exhibited the most competitive performance among the comparative algorithms. A significance level of α = 0.05 was adopted, and a *p*-value lower than 0.05 was considered to indicate a statistically significant difference in performance.

## 3. Results

### 3.1. Evaluation of Interpolation Methods and Data Density

**Figure 3:**
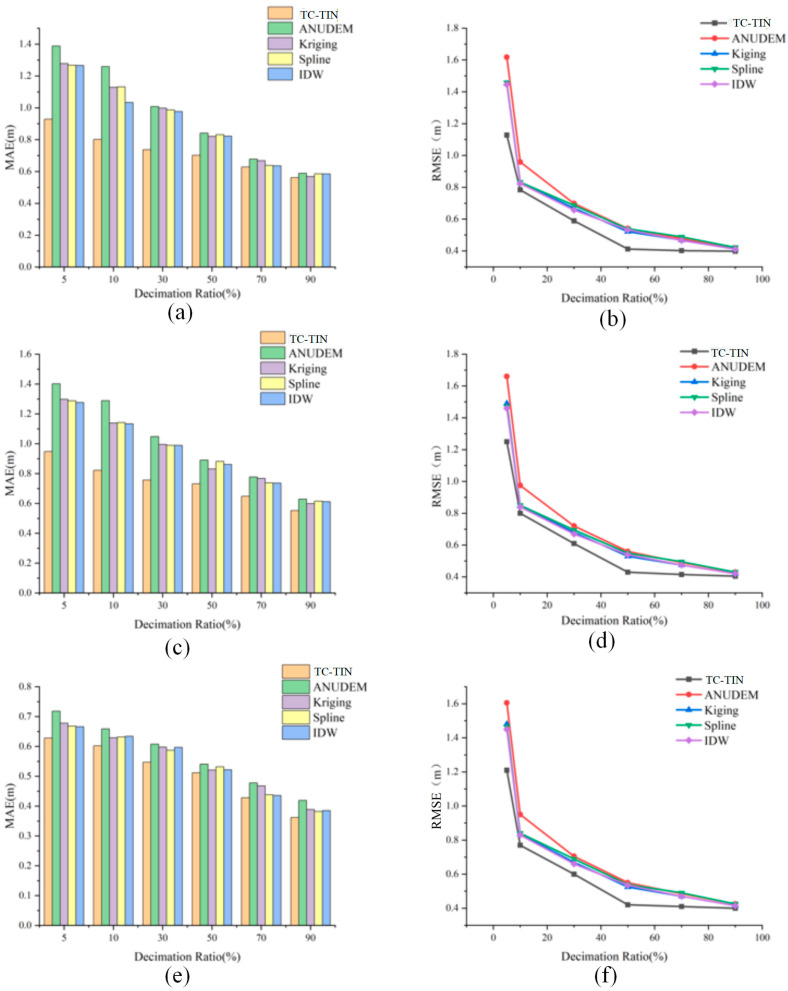
Relationship Between DEM Errors Generated by Different Interpolation Algorithms and Point Cloud Density: (**a**) Sample 1 MAE; (**b**) Sample 2 RMSE; (**c**) Sample 2 MAE; (**d**) Sample 2 RMSE; (**e**) Sample 3 MAE; (**f**) Sample 3 RMSE.

As illustrated in Figure 3, the DEM self-consistency results derived from different interpolation algorithms under five point cloud density conditions demonstrate a clear negative correlation between interpolation error and point density. With decreasing sampling rate, both MAE and RMSE values of all algorithms exhibit a consistent upward trend, indicating that sparser point clouds result in lower DEM self-consistency. This pattern is consistent across all three test areas, confirming that the influence of point density on interpolation self-consistency is universal. When the sampling rate is relatively high (≥70%), the differences in self-consistency among the five algorithms are minor; however, when it drops below 30%, the discrepancies become pronounced, revealing distinct variations in spatial adaptability among the algorithms.

Taking Test Area 3 (Figure 3e,f) as an example, when the sampling rate decreases from 90% to 10%, the RMSE of the proposed method increases moderately from approximately 0.41 m to 1.22 m, and the MAE from 0.353 m to 0.625 m, maintaining high self-consistency and stable interpolation performance under varying data densities. In contrast, ANUDEM and IDW exhibit a sharp increase in error as density decreases. Due to ANUDEM’s reliance on regular grid structures and hydrological consistency constraints, its local fitting capability is reduced when the data become sparse, leading to a rapid loss of self-consistency in complex mountainous terrain. Specifically, its RMSE rises from approximately 0.421 m to 1.613 m and MAE from 0.477 m to 0.767 m, showing a substantial decline in performance. The IDW method experiences the most significant degradation, with RMSE increasing from about 0.418 m to 1.432 m and MAE from 0.421 m to 0.761 m—corresponding to growth rates of 242.3% and 80.8%, respectively. Kriging and Spline maintain relatively stable self-consistency under medium to high density but deteriorate notably in sparse conditions, underscoring the limited spatial prediction capability of conventional interpolation methods.

Mechanistically, the proposed method effectively integrates terrain constraints with local spatial characteristics during interpolation. Through adaptive parameter adjustment, it dynamically responds to variations in topographic relief, mitigating over-smoothing and local distortion that often occur in traditional models. Consequently, even under sparse point cloud and complex terrain conditions, the method preserves DEM continuity and realism, demonstrating superior spatial adaptability and robustness to data sparsity. Overall, the proposed approach achieves higher DEM reconstruction self-consistency and stability across different point densities, offering a reliable and efficient solution for high-precision DEM generation in complex terrain environments.

The choice of a 10% sampling density for detailed visual and quantitative analysis stems from its representation of an extreme yet realistic UAV application scenario: encompassing the use of low-cost consumer drones, flying at higher altitudes to expand coverage, performing aggressive downsampling to enhance computational efficiency, or working with historical datasets where the original point cloud is inherently sparse. At this density—where the average point spacing increases by 8–10 times compared to the original—interpolation artifacts, such as “bull’s-eye” effects, excessive smoothing, and boundary discontinuities, become most pronounced. Consequently, this scenario maximally highlights differences in spatial adaptability and robustness among methods. In contrast, higher densities (≥50%) in complex terrain often mask algorithmic distinctions, whereas the 10% density vividly reveals performance contrasts with diagnostic value.

**Figure 4:**
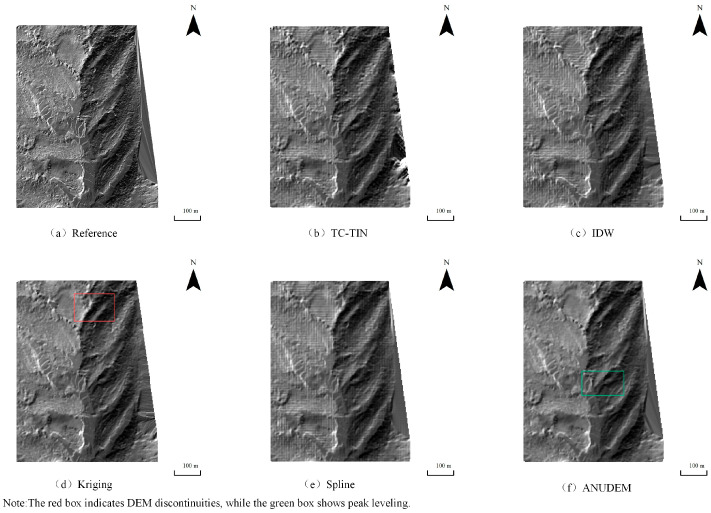
Hillshade Map of Test Area 1 Generated Under 10% Data Density Condition: (**a**) Hillshade generated from the reference DEM produced by Kriging using full-density data; (**b**) Hillshade generated by TC-TIN; (**c**) Hillshade generated by IDW interpolation; (**d**) Hillshade generated by Kriging interpolation; (**e**) Hillshade generated by Spline interpolation; (**f**) Hillshade generated by ANUDEM interpolation.

**Figure 5:**
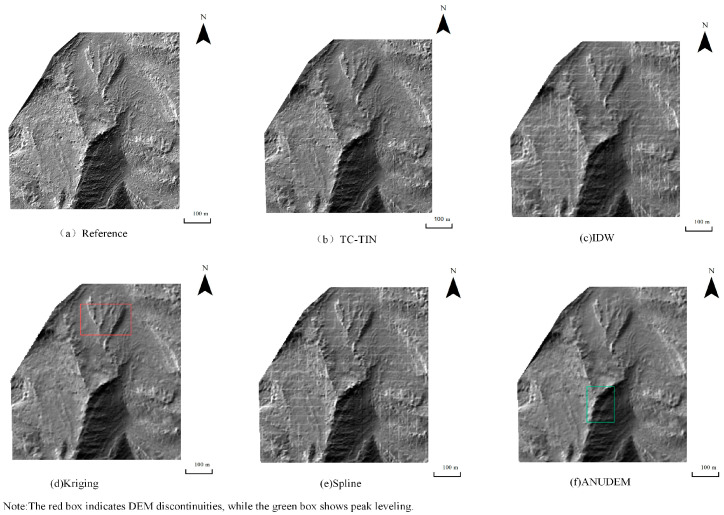
Hillshade Map of Test Area 2 Generated Under 10% Data Density Condition: (**a**) Hillshade generated from the reference DEM produced by Kriging using full-density data; (**b**) Hillshade generated by TC-TIN; (**c**) Hillshade generated by IDW interpolation; (**d**) Hillshade generated by Kriging interpolation; (**e**) Hillshade generated by Spline interpolation; (**f**) Hillshade generated by ANUDEM interpolation.

**Figure 6:**
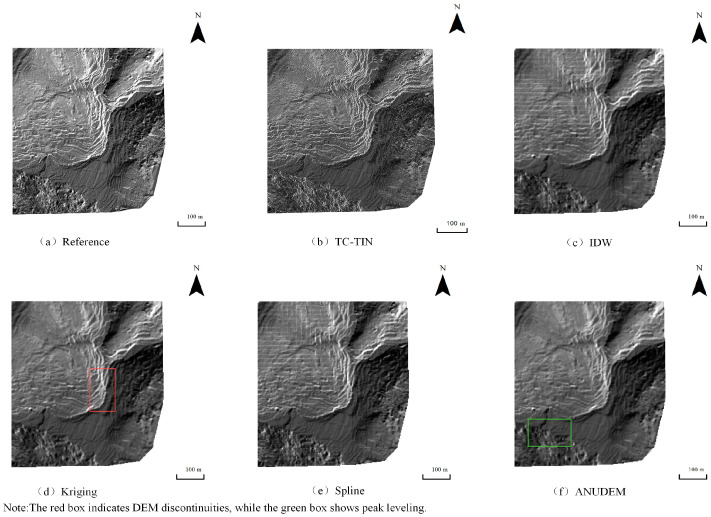
Hillshade Map of Test Area 3 Generated Under 10% Data Density Condition: (**a**) Hillshade generated from the reference DEM produced by Kriging using full-density data; (**b**) Hillshade generated by TC-TIN; (**c**) Hillshade generated by IDW interpolation; (**d**) Hillshade generated by Kriging interpolation; (**e**) Hillshade generated by Spline interpolation; (**f**) Hillshade generated by ANUDEM interpolation.

Under the 10% data density condition, the hillshade maps generated by the five interpolation algorithms are shown in Figure 4, Figure 5 and Figure 6. Using the DEM produced by Kriging under full-density data as the reference (Figure 4a, Figure 5a and Figure 6a), clear differences can be observed among the interpolation methods. The proposed method (Figure 4b, Figure 5b and Figure 6b) achieves favorable results even under low-density data, maintaining excellent surface continuity. ANUDEM interpolation (Figure 4f, Figure 5f and Figure 6f) tends to smooth terrain excessively due to its global smoothness assumptions, leading to flattened peaks in steep-slope areas and consequently reducing the realism of the reconstructed terrain. Kriging interpolation (Figure 4d, Figure 5d and Figure 6d) exhibits limited spatial inference capability and produces serrated, block-like artifacts along the boundaries, performing the worst overall. IDW interpolation (Figure 4c, Figure 5c and Figure 6c) is sensitive to sampling noise, resulting in rough surfaces with irregular bulges and poor robustness. In contrast, Spline interpolation (Figure 4e, Figure 5e and Figure 6e) demonstrates the most stable performance under low-density conditions, generating smooth and continuous surfaces that effectively preserve the overall terrain trend, particularly when sampling points are sparse.

Overall, the proposed method and the Spline interpolation perform best for terrain surface modeling and visualization under low data density conditions.

To provide a more objective and quantitative assessment, elevation difference maps were generated for Study Area 1. These newly added difference maps enable an intuitive, objective, and visually striking comparison: the proposed TC-TIN method consistently exhibits the largest near-white regions, with errors randomly distributed and no evident systematic bias. The Spline method performs robustly, ranking second, with smooth errors of relatively small magnitude. The IDW method shows noticeable artifacts. The Kriging method, affected by variogram instability in sparse data, displays pronounced stripe-like and grid-like errors. The ANUDEM method exhibits widespread peak clipping (deep blue negative bias) and valley filling (red positive bias).

### 3.2. Evaluation of Surface Roughness

Table 3 presents the RMSE values of surface roughness calculated by the proposed TC-TIN interpolation algorithm, Spline, Kriging, ANUDEM, and IDW across the three study areas under different point cloud densities (90%, 70%, 50%, 30%, 10%). Overall, as data density decreases, the RMSE values of all algorithms show an increasing trend, indicating a significant negative correlation between interpolation error and point cloud density. That is, sparser point clouds lead to lower self-consistency in DEM-based surface roughness estimation.

From the overall results, the proposed method demonstrates the lowest RMSE values and the smallest error increase across all study areas and density conditions, indicating its strong spatial adaptability and stability in complex terrain. The Spline and Kriging algorithms perform next best, showing relatively small errors under medium to high densities but experiencing rapid self-consistency degradation when data becomes sparse. ANUDEM consistently exhibits the highest RMSE values, with errors increasing significantly under low-density conditions, reflecting the weakening of terrain details caused by its “global smoothing assumption,” which tends to flatten peaks and raise valley bottoms. IDW shows relatively controllable errors under high density but is highly sensitive to uneven sampling, resulting in the most pronounced self-consistency drop when data density is insufficient.

In Study Area 1 (with significant terrain relief), the RMSE of the proposed method increases from 0.285 m to 0.358 m, a rise of approximately 25.6%, the smallest among all algorithms. In contrast, ANUDEM increases from 0.375 m to 0.508 m, a 35.5% increase. In Study Area 2 (moderate relief), the RMSE of the proposed method rises from 0.303 m to 0.366 m, an increase of 20.8%, significantly lower than that of ANUDEM (35.2%) and Kriging (31.6%). In Study Area 3 (relatively flat terrain), the overall RMSE values of all algorithms are the lowest. The proposed method achieves 0.219 m at 90% density and increases only to 0.259 m at 10% density, an 18.3% increase, demonstrating its high adaptability in flat terrain areas.

In summary, the RMSE of surface roughness systematically increases as point cloud density decreases, but the sensitivity of different algorithms to density changes varies significantly. The proposed method and Spline maintain high self-consistency and stability across both high and low densities, effectively preserving terrain details and roughness characteristics even in complex topography and under sparse point cloud conditions. In contrast, ANUDEM and Kriging, limited by their global smoothing and local weighting mechanisms, exhibit significant self-consistency degradation under low-density conditions and are unsuitable for high-precision terrain roughness estimation. Although IDW shows relatively small roughness RMSE values in several high-density cases (Table 3), its performance deteriorates rapidly under low-density conditions, which is consistent with its sensitivity to uneven point distribution. Therefore, despite its competitive results in some scenarios, IDW is less stable overall compared to the proposed method.

**Figure 7:**
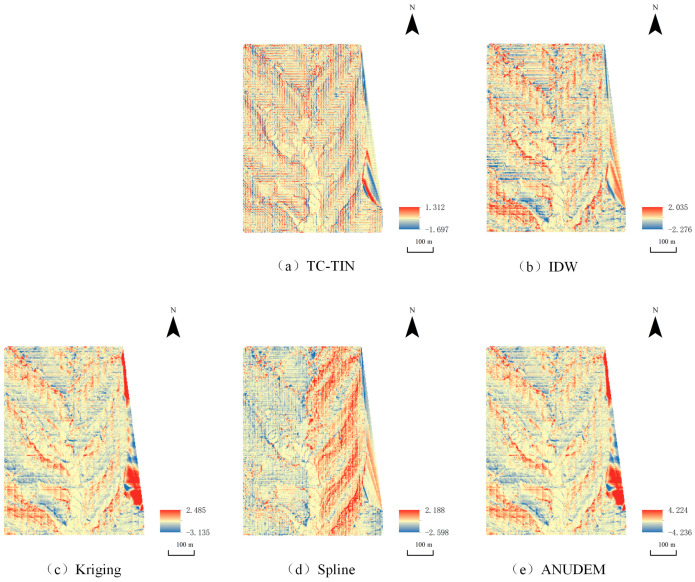
Comparison of interpolation errors at 10% point density for Study Area 1:The roughness map generated by Kriging interpolation under full-density conditions, serving as the reference benchmark; (**a**) The roughness map generated by TC-TIN; (**b**) The roughness map generated by IDW interpolation; (**c**) The roughness map generated by Kriging interpolation under low-density conditions; (**d**) The roughness map generated by Spline interpolation; (**e**) The roughness map generated by ANUDEM interpolation.

Taking the interpolation results under the 10% data density condition in Study Area 3 as an example, the corresponding surface roughness images are shown in Figure 7. The roughness map generated by Kriging interpolation under full density conditions (Figure 7a) serves as the reference benchmark. Overall, the performance of the various interpolation methods under this density shows notable differences.

Based on image tone and topographic structure, red areas primarily correspond to relatively gentle slopes and valleys, while green high-roughness zones are concentrated in steep slopes, fractured valleys, and areas surrounding hilltops, generally aligning with actual geomorphological features.

**Figure 8:**
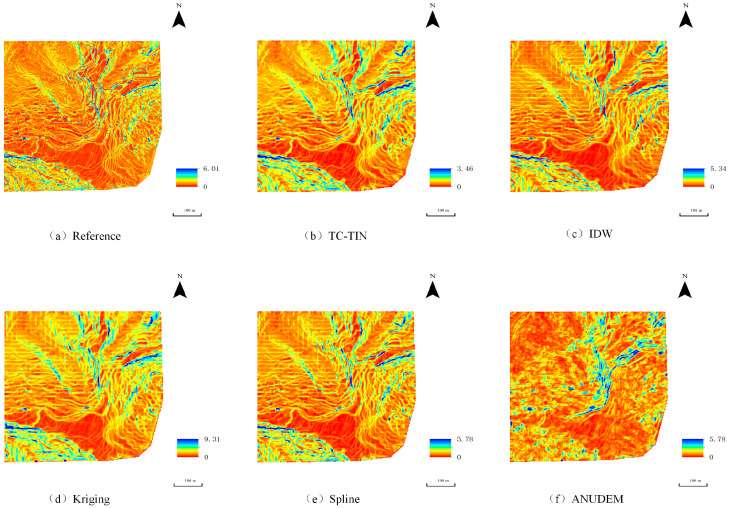
Surface roughness of the interpolated terrain in Test Area 3 under 10% point cloud density: (**a**) Reference roughness map generated by Kriging interpolation using full-density data; (**b**) Roughness map generated by TC-TIN interpolation method; (**c**) Roughness map generated by IDW interpolation; (**d**) Roughness map generated by Kriging interpolation under low-density conditions; (**e**) Roughness map generated by Spline interpolation; (**f**) Roughness map generated by ANUDEM interpolation.

The roughness maps generated by the proposed method (Figure 8b) and Spline interpolation (Figure 8e) most closely resemble the reference in terms of textural detail and tonal transition. The TC-TIN interpolation algorithm effectively preserves terrain undulations and micro-landform features, although minor discontinuities may occur in local areas due to fitting errors in the spatial variogram. The Spline interpolation produces a smoother roughness surface with more natural transitions, showing high consistency with the reference and demonstrating good stability and continuity.

In contrast, Kriging (Figure 8d) may introduce slight discontinuities in localized regions due to spatial variogram fitting inaccuracies. ANUDEM (Figure 8f) produces overly smoothed roughness results with poor representation of terrain details, while generating anomalously high values along edges due to the algorithm’s limited extrapolation capability. The roughness map generated by IDW interpolation (Figure 8c) contains multiple protruding areas, reflecting its susceptibility to discrete point noise and limited ability to represent topographic structures under low-density conditions.

### 3.3. Relative Difference Coefficient

To further quantitatively analyze the differences in surface reconstruction self-consistency among the various interpolation methods, this study employs the relative difference coefficient to conduct interactive comparisons of the DEMs generated by the five interpolation methods under the 10% point cloud density condition in Study Sample 1. One interpolation result is selected as the reference surface, while the other four are used as comparative subjects for interactive analysis. The formula for calculating the relative difference coefficient is as follows:

(5) $$\begin{array}{c} \mathrm{RDC}=1-\frac{\sum_{i=1}^{n}{|A}_{i}-B_{i}|}{\sum_{i=1}^{n}{|A}_{i}|} \end{array}$$

In this formula $A_{i}$ and $B_{i}$ represent the elevation values of the i-th pixel in the reference DEM and the comparative DEM, respectively, and n denotes the total number of pixels involved in the comparison. The numerator $\sum_{i=1}^{n}{|A}_{i}-B_{i}|$ measures the cumulative absolute elevation difference between the two datasets, while the denominator $\sum_{i=1}^{n}{|A}_{i}|$ normalizes this difference by the total magnitude of the reference DEM.

The RDC value ranges from negative values to 1. A value closer to 1 indicates a higher degree of similarity between the two DEMs. Values approaching 0 represent substantial discrepancies, and values less than 0 imply that the difference between the datasets exceeds the overall magnitude of the reference DEM, suggesting strong inconsistency or non-comparability. This metric provides a normalized and intuitive assessment of DEM similarity suitable for evaluating interpolation results under varying point cloud densities.

As shown in Table 4, the relative difference coefficients between TC-TIN interpolation algorithm and other methods are generally high. Specifically, the coefficient with Spline interpolation reaches 0.9991, the highest among all combinations, indicating the closest agreement in terms of generated DEM surface morphology, slope continuity, and elevation trends. In contrast, the coefficients with Kriging and ANUDEM are 0.9986 and 0.9983, respectively, slightly lower than the former, suggesting minor deviations in areas with abrupt elevation changes or steep slopes, which result in slightly weaker surface continuity.

In summary, under low-density point cloud conditions, the TC-TIN interpolation algorithm demonstrates the closest alignment with Spline results, exhibiting optimal spatial consistency and local self-consistency. It effectively restores terrain details while ensuring DEM continuity, highlighting its strong spatial adaptability and stability.

### 3.4. Statistical Significance of Interpolation Differences

To determine whether the performance differences between the proposed method and the comparative algorithms are statistically significant, paired t-tests were conducted using RMSE and MAE values across all study areas and sampling densities. Table 5 summarizes the results.

## 4. Discussion

The validation relies solely on cross-validation of UAV-derived ground points, which cannot represent true absolute accuracy. Thus, the RMSE/MAE values reported here reflect interpolation fidelity rather than ground-truth errors. This is a major limitation and future studies should incorporate TLS or RTK survey benchmarks.

### 4.1. Interpretation of Key Findings

The superior performance of the proposed TC-TIN method can be attributed to its effective integration of terrain feature constraints and adaptive point selection mechanism. Unlike global smoothing methods like ANUDEM, which tend to oversimplify complex terrain features, our approach preserves critical topographic characteristics through constrained Delaunay triangulation. The significant correlation (RDC > 0.999) between our method and Spline interpolation under low-density conditions suggests both methods effectively maintain surface continuity, though through different mathematical frameworks.

Although the proposed TC-TIN method demonstrates relatively stable performance, Spline interpolation consistently shows strong competitiveness, particularly under low-density conditions. Its ability to preserve global smoothness and maintain surface continuity makes Spline a robust baseline method in complex terrain. In several cases, the performance of Spline approaches or exceeds that of the proposed method, which highlights the importance of treating the TIN-based refinements as a comparative improvement rather than a universally superior solution.

### 4.2. Comparison with Existing Methods

The performance variations among different interpolation methods reflect their inherent algorithmic characteristics. ANUDEM’s poor performance in complex terrain aligns with previous studies [27,28], confirming its limitations in handling abrupt elevation changes. The sensitivity of IDW to sampling density, particularly evident in its 34.1% RMSE increase from 90% to 10% density, underscores its dependence on point distribution uniformity. Kriging’s intermediate performance suggests that while its geostatistical framework provides theoretical advantages, its practical effectiveness is constrained by variogram fitting self-consistency in data-sparse scenarios.

### 4.3. Implications for DEM Generation in Complex Terrain

Our results demonstrate that successful DEM generation in complex mountainous areas requires algorithms capable of adapting to spatially variable terrain complexity; preserving breaklines and feature edges; and maintaining stability across different data densities. The proposed method addresses these requirements through its terrain-constrained approach and robust point selection strategy, achieving <25% error increase even at 10% sampling density.

### 4.4. Limitations and Research Directions

While the proposed method shows significant advantages, several aspects warrant further investigation: the computational efficiency compared to simpler methods like IDW needs optimization for large-scale applications; the method’s performance in extremely flat terrain requires additional validation; and integration with deep learning approaches could potentially enhance parameter optimization and feature recognition. Future research should also explore the method’s applicability to different sensor data and various landscape types beyond the tested mountainous terrain.

## 5. Conclusions

This study, focusing on the southern mountainous area of the Lufeng Dinosaur National Geopark in Yunnan Province, utilized high-density point cloud data acquired by UAVs to systematically analyze the DEM generation self-consistency and surface roughness response characteristics of different spatial interpolation methods under various sampling densities. An improved TC-TIN interpolation algorithm was proposed. The main conclusions are as follows:

(1) As the point cloud sampling rate decreases, the self-consistency of DEMs generated by all interpolation algorithms shows a declining trend, with corresponding increases in RMSE and MAE values. A significant negative correlation exists between data density and interpolation error. DEM self-consistency remains relatively stable when the point cloud density is above 50% of the original data; however, when the density drops below 30%, errors increase sharply, particularly for the IDW and ANUDEM methods, which exhibit the most pronounced decline in self-consistency.
(2) Traditional ANUDEM and IDW methods are prone to generating smoothing effects or distortions under sparse data conditions. Spline interpolation demonstrates good smoothness and continuity under low-density conditions, effectively preserving general terrain trend characteristics. In contrast, the proposed TC-TIN interpolation method exhibits strong spatial stability and self-consistency retention capability under both high- and low-density conditions. It achieves the lowest RMSE growth rate and effectively suppresses edge discontinuities and error accumulation.
(3) Surface roughness is closely related to DEM self-consistency. Roughness increases with greater terrain relief, and in areas of high roughness, all interpolation algorithms generally exhibit larger errors. The proposed method maintains low error levels across different roughness grades, indicating that the terrain-constrained mechanism enhances the interpolation’s adaptability to complex surface undulations to a certain extent.
(4) The improved TC-TIN interpolation algorithm possesses strong general applicability and practical value. Through the seed point screening and boundary auxiliary point introduction mechanisms, this method effectively eliminates interference from non-ground points, optimizes the TIN mesh structure, enhances the spatial continuity and terrain authenticity of the DEM while maintaining computational efficiency, making it particularly suitable for DEM reconstruction tasks in complex mountainous areas and under sparse point cloud conditions.

In summary, the research results validate the feasibility and comparative analysis of the TC-TIN interpolation algorithm under complex terrain conditions, providing theoretical support and technical reference for generating high-precision DEMs from UAV point clouds and for subsequent terrain factor analysis. Future research could build upon this work by further incorporating terrain zoning characteristics or introducing deep learning models to achieve adaptive optimization of interpolation parameters and intelligent enhancement of DEM self-consistency.

## Figures and Tables

**Table 1:** Statistical Information of the Experimental Data.

Study Area	Area/m^2^	Elevation Range/m	Mean Elevation/m	Point Cloud Density (pts/m^2^)
Sample 1	354,534.61	1591.86–1687.47	1640.71	1.49
Sample 2	409,610.48	1596.47–1744.77	1669.82	1.49
Sample 3	348,367.81	1530.51–1631.62	1568.82	1.68

**Table 2:** Number of Point Clouds After Reduction in the Test Area.

	Number of Points/pcs					
Study area	100%	90%	70%	50%	30%	10%
Sample 1	529,319	476,387	370,523	264,659	158,795	52,931
Sample 2	610,167	549,150	427,116	305,083	183,050	61,016
Sample 3	585,514	526,962	409,859	292,757	175,654	58,551

**Table 3:** RMSE of Surface Roughness Calculated by Different Algorithms.

Study Area	Data Density (%)	RMSE (m)				
		TC-TIN	Spline	Kriging	ANUDEM	IDW
Sample 1	90	0.285	0.310	0.358	0.375	0.295
	70	0.298	0.311	0.365	0.382	0.298
	50	0.307	0.323	0.390	0.416	0.322
	30	0.314	0.325	0.428	0.460	0.355
	10	0.358	0.367	0.472	0.508	0.392
Sample 2	90	0.303	0.319	0.361	0.378	0.294
	70	0.317	0.324	0.368	0.384	0.297
	50	0.338	0.352	0.393	0.420	0.325
	30	0.346	0.364	0.430	0.463	0.358
	10	0.366	0.374	0.475	0.511	0.394
Sample 3	90	0.219	0.230	0.235	0.248	0.205
	70	0.230	0.241	0.239	0.252	0.207
	50	0.239	0.247	0.258	0.267	0.220
	30	0.247	0.264	0.277	0.287	0.238
	10	0.259	0.287	0.308	0.312	0.260

**Table 4:** Relative Difference Coefficient of Each Interpolation Method.

Area	Comparison of Interpolation Methods	Benchmark Interpolation Method				
		TC-TIN	Spline	Kriging	ANUDEM	IDW
Sample 1	Ours	——	0.9991	0.9986	0.9983	0.9988
	Spline	0.9991	——	0.9985	0.9981	0.9986
	Kriging	0.9986	0.9985	——	0.9979	0.9982
	ANUDEM	0.9983	0.9981	0.9979	——	0.9980
	IDW	0.9988	0.9986	0.9982	0.9980	——

**Table 5:** Results of Paired t-test for RMSE Differences.

Comparison	Mean RMSE Difference (m)	*p*-Value	Significance
Proposed vs. Spline	−0.021	0.013	Significant (*p* < 0.05)
Proposed vs. Kriging	−0.048	0.005	Significant (*p* < 0.05)

## Data Availability

The data presented in this study are available from the corresponding author upon reasonable request.
